# Tailoring Low-Temperature Tempering to Dramatically Enhance Compressive Ductility and Fatigue Contact Wear Resistance in High-Carbon Bearing Steel

**DOI:** 10.3390/ma19153343

**Published:** 2026-08-06

**Authors:** Hui Li, Xiangkun Song, Qing Tao, Zhenqian Wang, Qiulai Huang, Weipeng Xu, Qingliang Li, Jian Wang

**Affiliations:** 1 Shanghai, TianDi Mining Equipment Technology Co., Ltd., Shanghai 200030, China; 2 School of Mechanical and Electrical Engineering, China University of Mining and Technology, Xuzhou 221116, China; 3 State Key Laboratory of Intelligent Mining Equipment Technology, China University of Mining and Technology, Xuzhou 221116, China

**Keywords:** high-carbon martensite steel, low-temperature tempering, mechanical properties, fatigue contact, wear resistance

## Abstract

High-carbon martensitic steels for bearing components are conventionally low-temperature tempered for stress relief, yet the influence of tempering temperature on compressive and fatigue wear resistance remains unclear, directly affecting the service life of bearing races and rollers. In this study, a high-carbon martensitic steel was tempered at 170 °C, 200 °C, and 230 °C. The microstructural evolution, compressive properties, and contact fatigue wear resistance were systematically investigated, along with the corresponding strengthening and wear mechanisms. After spheroidizing annealing and quenching, the microstructure consists of high-carbon martensite and retained austenite, with a high density of dislocations and fine twins. Tempering decomposes retained austenite into tempered martensite and promotes fine carbide precipitation, processes that become more pronounced at higher temperatures. Consequently, hardness decreases from 810 HV in the as-quenched state to 690 HV after 230 °C tempering, while compressive failure strain increases from 10.5% to 24.1%. More importantly, under cyclic contact stress, the 230 °C-tempered specimen exhibits approximately 33% lower wear mass loss than the 170 °C-tempered counterpart, despite its lower hardness. This unexpected improvement is attributed to the formation of a distinct plastic deformation zone in the near-surface region, which absorbs greater strain energy and delays fatigue spallation. The well-tempered martensitic matrix accommodates more long-range dislocation slip, enabling a transition from fatigue spallation to a more ductile failure mode. These findings provide new insights into the role of low-temperature tempering in balancing strength, ductility, and wear resistance, and offer practical guidance for optimizing heat treatment protocols to enhance the contact fatigue performance of bearing steels.

## 1. Introduction

High-carbon steels are widely utilized in the manufacturing of high-performance bearing components [1]. Compared to the low- and medium-carbon steels commonly employed for structural parts, shafts, and gears [2], high-carbon bearing steels achieve their strength primarily through solid solution strengthening enabled by a carbon content of approximately 1 wt.%, with strengths typically exceeding 2.0 GPa [3,4]. During service, these components are predominantly subjected to high, cyclically applied compressive and contact stresses. Under prolonged loading, fatigue microcracks tend to initiate at subsurface regions of maximum stress [5,6,7], which can further propagate, leading to fatigue spalling and compromising bearing operational stability.

The initiation of fatigue microcracks originates from several factors, including stress concentration sites from martensitic transformation, interfaces between metallurgical inclusions and the matrix, and regions of carbide agglomeration. Furthermore, within the affected zone of subsurface contact fatigue stress, so-called white etching areas (WEAs) [8,9,10] readily form and can subsequently develop into fatigue crack nuclei. These WEAs result from localized microstructural shear deformation, which promotes the accumulation of high-density dislocations and leads to grain refinement or nano-crystallization. This process increases local hardness and brittleness, ultimately facilitating micro-crack formation. However, it is undeniable that during the formation and propagation of fatigue microcracks, localized plastic deformation inevitably occurs in the immediate vicinity due to dislocation slip [6,11]. Consequently, a key approach to enhancing the contact fatigue resistance of high-carbon bearing steels is to improve the microstructural resistance to dislocation slip.

For high-carbon bearing steels, the typical microstructure consists of high-carbon martensite after quenching [12,13] or high-carbon bainite formed through isothermal quenching [14], both containing a small amount of retained austenite. However, it is widely recognized that directly quenched high-carbon martensite is a characteristically brittle microstructure. The strong pinning of mobile dislocations by carbon atoms inhibits the transformed phase from accommodating strain via dislocation slip, resulting in plate-like martensite with a twinned substructure and exceptionally poor ductility and toughness [3,15]. Consequently, a spheroidizing annealing treatment is commonly applied prior to quenching [16]. This process promotes the precipitation of carbon as numerous granular carbides. During subsequent austenitization in the quenching heating stage, a portion of these granular carbides remains undissolved, thereby diluting the carbon content in the matrix [17,18,19] and ultimately reducing the internal stress of the quenched martensite.

Nevertheless, although retained austenite has long been regarded as an unstable, detrimental phase whose content is typically minimized, a substantial body of research suggests its potential can be positively utilized. A representative approach is the Quenching and Partitioning (Q&P) process [20], which retains a limited amount of austenite and enhances its mechanical stability—specifically its resistance to transformation (anti-TRIP capability)—through carbon partitioning, thereby achieving improved mechanical properties [21,22].

Furthermore, low-temperature tempering can significantly mitigate the brittleness of high-carbon martensite [3]. This improvement stems from two primary mechanisms: firstly, it relieves the lattice strain induced by the martensitic transformation; secondly, it promotes the precipitation of transitional carbides [23,24]. These carbides enable dislocation slip within the martensitic matrix under external stress, thereby enhancing ductility and toughness. In practice, bearing steels of this type are typically tempered at approximately 150–180 °C, while their in-service operating temperatures may be about 30 °C higher due to frictional heating [1]. Existing research, including our prior work [4], demonstrates that tempering temperatures in the range of 175–250 °C significantly affect the strength and ductility of high-carbon martensitic steel, with tensile strength reaching 2.5–2.7 GPa and elongation approximately 2%. However, these studies have primarily focused on conventional mechanical properties, and few have investigated the influence of low-temperature tempering on the contact fatigue wear performance of high-carbon martensitic steels, a critical determinant of the service life of bearing races and rollers.

Accordingly, this research aims to systematically clarify the effects of low-temperature tempering at 170 °C, 200 °C, and 230 °C on the mechanical properties and contact fatigue wear behavior of a high-carbon martensitic bearing steel. The work involves a systematic evaluation of microstructural evolution, compressive properties, and contact fatigue performance. Specific analyses focus on the morphology of martensite and tempered martensite, along with the content of retained austenite. Furthermore, the strengthening-toughening and wear resistance mechanisms associated with different tempering treatments are discussed.

## 2. Materials and Methods

**Figure 1:**
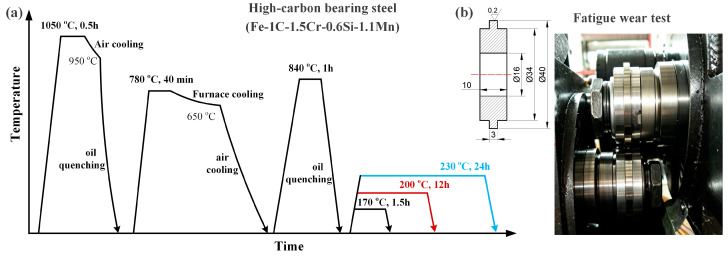
(**a**) Schematic of the heat treatment process for the high-carbon martensitic steel; (**b**) sample geometry and test setup for contact fatigue testing.

The high-carbon martensitic steel used in this study was a hot-rolled and normalized bearing steel with a nominal composition of Fe-1C-1.5Cr-0.6Si-1.1Mn. As illustrated in Figure 1a, the hot-rolled and normalized slab was subjected to homogenization annealing at 1050 °C, followed by air cooling to 950 °C and subsequent oil quenching. The slab was then reheated to 780 °C, held for 40 min, and then furnace-cooled at a rate of approximately 0.4 °C/min to 650 °C with a total cooling time of about 5 h, and finally air-cooled to room temperature. This slow cooling stage, combined with the preceding isothermal hold, promotes the divorced eutectoid transformation and the development of a spheroidized carbide microstructure.

After this pretreatment, the slab was turned from approximately Φ50 mm to the final dimension of Φ42 × 100 mm to remove any surface layer that may have been affected by decarburization during the preceding heat treatments. To prevent surface decarburization during subsequent heating, the specimens were austenitized at 840 °C for 1 h in a vacuum furnace, followed by oil quenching. The quenched samples were sectioned and subjected to low-temperature tempering at 170 °C for 1.2 h, 200 °C for 12 h, and 230 °C for 24 h, respectively. This approach was designed to investigate the differential effects of various tempering parameters on the microstructure and properties of the quenched high-carbon martensite.

The microstructural features of pearlite, martensite, and tempered martensite under different processing conditions were characterized using scanning electron microscopy (SEM. TESCAN, Brno, Czech Republic) and transmission electron microscopy (TEM, Tecnao G2 F20, Stanford, CA, USA). All SEM and TEM images presented in this study are representative of the microstructural features observed across multiple regions of the specimens. The variation in the volume fraction of retained austenite in the microstructure after quenching and subsequent low-temperature tempering was analyzed by X-ray diffraction (XRD, Bruker, Berlin, Germany). For SEM examination, specimens were mechanically polished and etched with a 4 vol.% nital solution. TEM samples were mechanically ground to approximately 80 µm in thickness and then thinned to electron transparency by twin-jet electro-polishing using a 10 vol.% perchloric acid ethanol solution at 30 V and −20 °C. Specimens for XRD analysis were mechanically ground and electrolytically polished with a 5 vol.% perchloric acid ethanol solution at 20 V.

Compressive mechanical properties of specimens prepared under different conditions were evaluated using a CMT5505 universal testing machine (SUNS, Shenzhen, China). The compression specimens, measuring Φ4 × 10 mm, were tested at a strain rate below 1 × 10^−3^ s^−1^, with three replicate specimens tested for each condition. Hardness measurements were conducted on a THV-1MDT micro-mechanical property tester (Time Shijin Testing Machine, Jinan, China) with a load of 1 kgf and a dwell time of 10 s. For each specimen, five indentations were made, and the average value was used for subsequent analysis, resulting in at least 15 individual measurements per condition. The hardness data are presented as the mean ± standard deviation (SD) to reflect data dispersion. Contact fatigue wear tests were performed on an M2000 tester (Jingcheng, Jinan, China). The specimen geometry is shown in Figure 1b. The tests were conducted under a normal load of 1500 N at a rotational speed of 400 rpm, with three specimens tested per tempering condition. After testing, specimens were weighed to determine mass loss. Subsequently, they were sectioned to measure the hardness gradient along the longitudinal cross-section and to observe the microstructural characteristics adjacent to the wear surface.

## 3. Results and Discussion

### 3.1. Microstructure and Mechanical Properties

**Figure 2:**
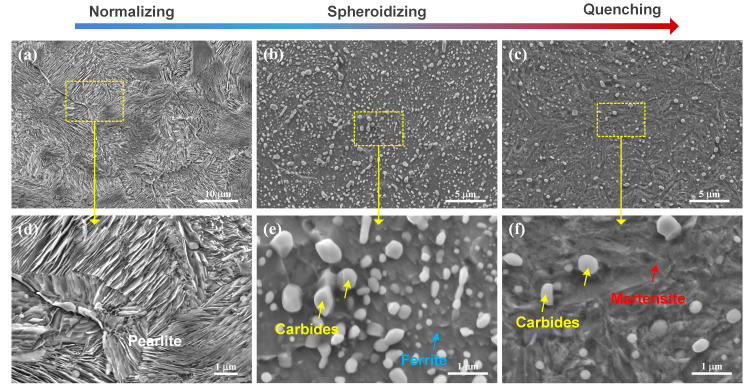
Microstructure from SEM of the samples treated by normalizing (**a**), spheroidizing (**b**), and quenching (**c**–**f**) are the enlarged views in (**a**–**c**). After quenching, ferrite matrix has been transformed into high-carbon martensite.

Given that the carbon content of the adopted high-carbon bearing steel exceeds the eutectoid critical value, its microstructure after hot rolling and normalizing (slow cooling) consists predominantly of pearlite, with some intergranular carbides distributed along the prior austenite grain boundaries, as shown in Figure 2a. If quenching were performed directly after normalizing, the lamellar pearlite within the original austenite grains would dissolve rapidly during re-austenitization [25,26], leading to a sharp increase in austenite carbon content [27]. The subsequent quenching would then produce plate martensite, increasing brittleness. Furthermore, the carbides at the original austenite grain boundaries dissolve more slowly [28], and their persistence can also lead to brittle intergranular cracking. Therefore, a high-temperature homogenization solution treatment followed by spheroidizing annealing is essential to promote the uniform precipitation of carbides within the matrix, as depicted in Figure 2b. The strength after spheroidizing annealing decreases, facilitating subsequent machining operations.

**Figure 3:**
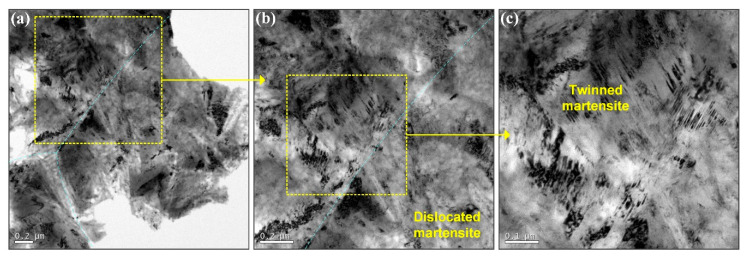
(**a**) TEM analysis of the quenched samples. (**b**,**c**) are the enlarged views. The dislocations and twinned martensite can be observed, indicating high carbon content in martensite matrix.

The microstructural features after reheating to 840 °C followed by oil quenching are shown in Figure 2c. A portion of incompletely dissolved granular carbides is retained, while the finer carbide particles have fully dissolved. Concurrently, the matrix transforms from the ferritic structure present after spheroidizing annealing to martensite. No quench-induced microcracks were observed. Further analysis of the matrix microstructure by transmission electron microscopy (TEM) is presented in Figure 3. The images reveal characteristics typical of high-carbon martensite. Its substructure contains both a high density of dislocations and fine twins, indicating the presence of substantial internal stresses, which necessitates subsequent low-temperature tempering treatment.

The high density of dislocations and twinned substructure in the as-quenched high-carbon martensite has been widely documented in 52100-type bearing steels [1]. These crystal defects provide abundant nucleation sites for carbide precipitation during subsequent tempering [29]. Regarding the content of retained austenite, it has been reported that after austenitization at approximately 840 °C followed by quenching, the retained austenite content in 52100 steel is typically around 20%. In the present study, the measured value is approximately 15.5%, slightly lower than this typical level, which may be attributed to differences in the alloy composition of the experimental steel.

**Figure 4:**
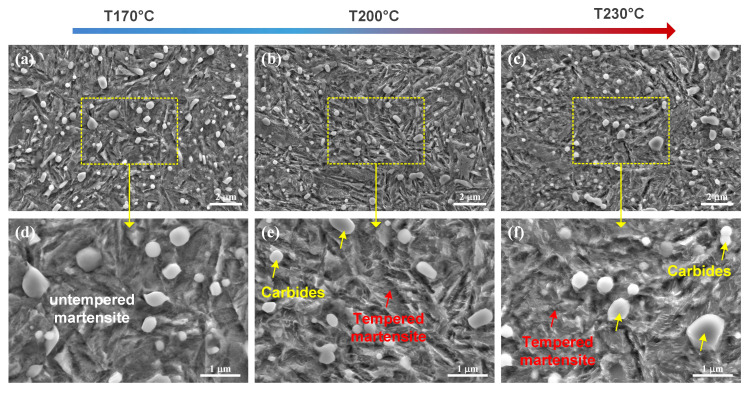
Microstructure from SEM of the samples treated by normalizing, spheroidizing, quenching, and tempering at 170 °C (**a**), 200 °C (**b**), and 230 °C (**c**–**e**), and (**f**) is the enlarged view in (**a**–**c**).

**Figure 5:**
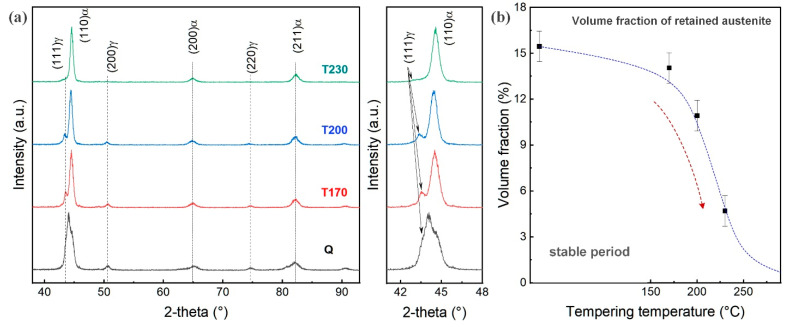
XRD profiles (**a**) and volume fraction of retained austenite (**b**) for the samples after quenching and tempering at 170 °C, 200 °C, and 230 °C.

The microstructural evolution of specimens after low-temperature tempering is shown in Figure 4. Following tempering at 170 °C, the matrix largely retains the martensitic morphology of the as-quenched state, with no significant carbide precipitation observed. At this temperature, the primary effect on the quenched martensite is the stabilization of lattice dislocations induced by the phase transformation, which promotes the relief of internal stresses. Additionally, carbon atom clustering could occur within localized micro-regions, acting as precursors to transition carbides [30,31]. Furthermore, as indicated in Figure 5, the retained austenite content decreases from approximately 15.5% in the as-quenched condition to about 14% after tempering. This suggests that a tempering temperature of 170 °C provides insufficient driving force for extensive decomposition of the retained austenite.

Concerning the decomposition behaviour of retained austenite in high-carbon steels during tempering, it has been reported that the decomposition of retained austenite is not significant at tempering temperatures around 160 °C; only when the tempering temperature approaches or exceeds 200 °C does the decomposition rate become appreciably accelerated [3,4]. In this study, the retained austenite content changed only marginally after tempering at 170 °C, which is consistent with this temperature threshold. Moreover, it is well established that during low-temperature tempering below 200 °C, the primary changes in quenched martensite involve the stabilization of the dislocation structure and the relief of quenching-induced internal stresses, rather than significant carbide precipitation. The absence of observable carbides after tempering at 170 °C in the present work is in agreement with this understanding.

With increasing tempering temperature, fine carbides can precipitate within the martensitic matrix, becoming more pronounced at 230 °C, as shown in Figure 4c,f. The carbides formed during low-temperature tempering are predominantly transition carbides [32,33], uniformly distributed inside the martensite laths or plates. Consequently, the solid-solution carbon content in the original martensite matrix is diluted, leading to a reduction in solid-solution strengthening and a decrease in the stored dislocation density [4]. At this tempering temperature, grain boundary migration is not activated, and the matrix retains the interfaces of the martensite laths and plates. Figure 5 presents the XRD results tracking the evolution of retained austenite with tempering temperature. For the as-quenched specimen, the diffraction peaks near 45° exhibit pronounced broadening rather than discrete reflections. This is characteristic of high-carbon martensite, whose body-centered tetragonal (BCT) lattice gives rise to closely spaced (110) and (101) peaks that overlap with each other and with the retained austenite reflection, making the latter appear indistinct. After tempering, however, the tetragonality decreases as carbon leaves the martensite lattice, and the peaks become better resolved, allowing the retained austenite content to be reliably quantified. As shown in Figure 5, the retained austenite content gradually decreases with higher tempering temperatures. Its decomposition products, which can be regarded as tempered martensite or lower bainite [34], show little microstructural distinction from the surrounding matrix.

**Figure 6:**
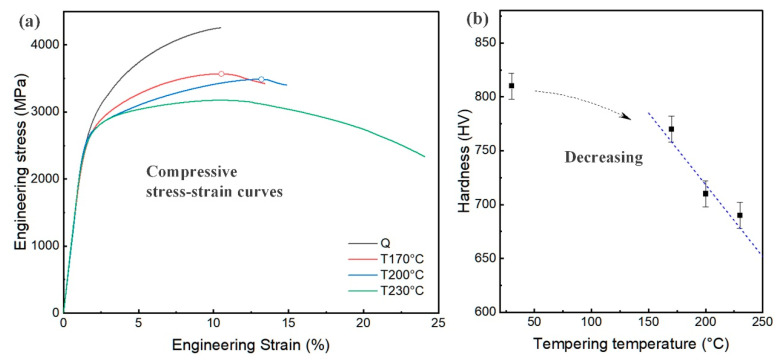
Compressive stress–strain curves (**a**) and hardness (**b**) for the samples after quenching and tempering at 170 °C, 200 °C, and 230 °C.

The compressive stress–strain curves and microhardness evolution of the quenched and tempered samples at different temperatures are presented in Figure 6 and Table 1. As shown, although the ultimate compressive strength of the as-quenched sample exceeds 4000 MPa, its compressive failure strain is only about 10%. After tempering at 170 °C, the ultimate strength is 3566 MPa, while the compressive failure strain increases to 13.5%. With a further increase in tempering temperature, a compressive failure strain of 24% can be achieved, while the ultimate strength remains at approximately 3100 MPa.

It should be noted that the strain hardening rate under compression also decreases with increasing tempering temperature, which is closely related to the differences in the underlying strengthening and toughening mechanisms within the microstructure. This variation in the strain hardening response under compressive stress implies that there must be differences in their contact fatigue performance during service. Correspondingly, the microhardness gradually decreases from about 800 HV in the as-quenched state to around 700 HV after tempering.

### 3.2. Fatigue-Wear Behavior

**Figure 7:**
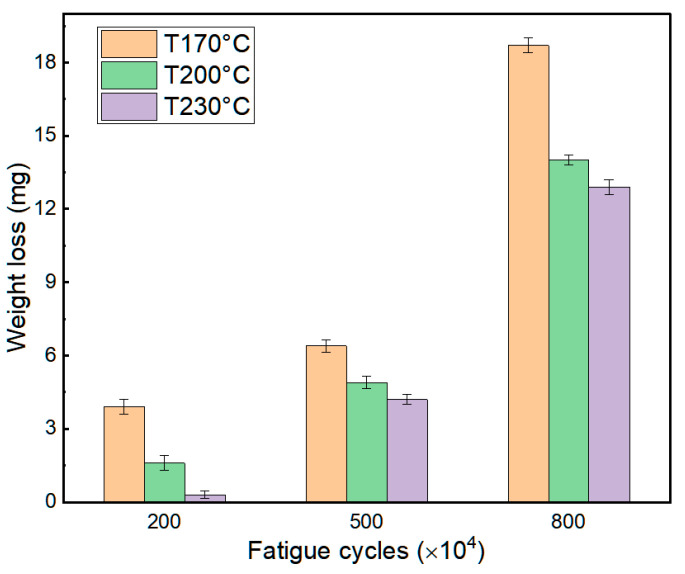
Weight loss of the fatigue wear-tested samples with different tempering at 170 °C, 200 °C, and 230 °C.

Resistance to contact fatigue wear is the most critical performance indicator for high-carbon bearing steels [19,35,36], typically evaluated by the mass loss after millions of cycles. Although directly quenched samples can achieve high strength and hardness, internal quenching stresses, unstable dislocations, and retained austenite can adversely affect operational stability during contact [1]. Consequently, high-carbon bearing steels for practical applications must undergo low-temperature tempering to varying degrees. Accordingly, we measured the fatigue wear mass loss of samples tempered at different temperatures. It should be noted that the present study focuses on the wear mass loss at fixed fatigue cycles (2, 5, and 8 million cycles) to evaluate the effect of tempering temperature on wear resistance, rather than on the statistical distribution of fatigue life. Therefore, Weibull distribution analysis, which is typically employed for fatigue life characterization, was not applied in this work. The results are presented in Figure 7.

As shown, the wear mass loss exhibits an exponential increase with the number of contact cycles for all conditions. In comparison, the sample tempered at 230 °C shows the lowest wear mass loss, approximately 12 mg after 8 million cycles. Under the same cyclic loading, however, the wear mass loss of the sample tempered at 170 °C exceeds 18 mg. Conventional theory generally posits that higher hardness correlates with better wear resistance. The present results, therefore, are unexpected, indicating that samples with relatively lower hardness exhibit superior wear resistance.

This result contradicts the conventional expectation that higher hardness invariably confers better wear resistance. It is noteworthy that the contact fatigue behaviour of bearing steels is not governed solely by hardness. Previous studies have clearly demonstrated that the ductility and toughness of tempered martensite exert a non-negligible influence on rolling contact fatigue resistance, as improved plasticity and toughness [1,3,37] enable the material to dissipate contact strain energy through localized plastic deformation, thereby retarding fatigue crack initiation and propagation. On this basis, the anomalous observation can be rationalized as follows: although the 170 °C-tempered specimens possess higher hardness, the high solid-solution carbon content in the martensitic matrix severely pins dislocation motion, making it difficult to accommodate strain through dislocation slip under cyclic contact stress; consequently, damage is dominated by brittle spallation. In contrast, although the 230 °C-tempered specimens exhibit lower hardness, the more complete precipitation of carbides improves the ductility and toughness of the matrix, allowing a certain degree of plastic deformation to occur in the near-surface region under cyclic contact stress. This enables greater absorption of contact strain energy and delays the onset of fatigue spallation, ultimately resulting in lower wear mass loss.

**Figure 8:**
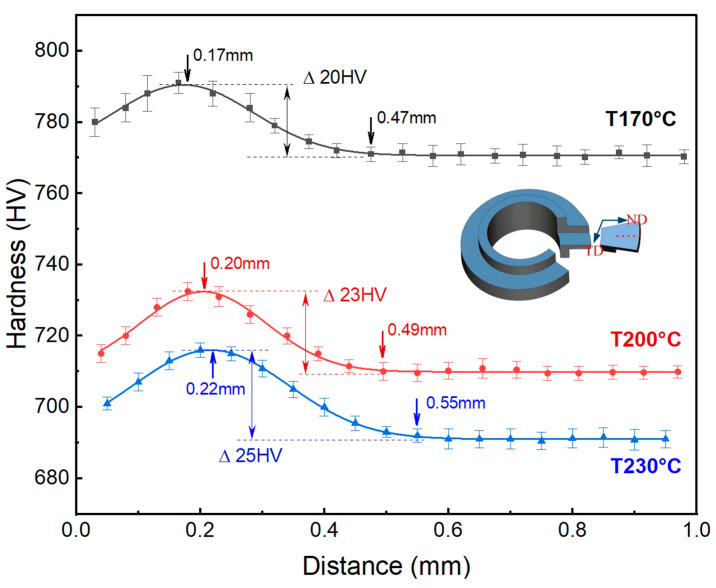
Microhardness distribution along normal direction of the fatigue wear-tested samples with different tempering at 170 °C, 200 °C, and 230 °C.

To further clarify this anomalous trend, the hardness distribution across the cross-section of samples subjected to 5 million contact fatigue cycles was analyzed. Figure 8 shows the microhardness profiles along the depth direction for samples treated at different tempering temperatures. A distinct trend is observed: within a certain depth below the contact surface, the microhardness initially increases and then decreases, with the peak hardness occurring in the subsurface region. This peak hardness is approximately 20 HV higher than the bulk hardness, corresponding to the location of maximum contact stress. The depth of this work-hardened zone—where hardness exceeds the bulk value—extends to about 0.5 mm.

The key distinction, however, lies in the extent to which cyclic contact stress alters the hardness distribution for samples tempered at different temperatures. Specifically, with increasing tempering temperature, the magnitude of the subsurface hardness increase rises (from Δ20 HV to Δ25 HV), the depth corresponding to the peak hardness increases (from 0.17 mm to 0.22 mm), and the overall affected depth expands (from 0.47 mm to 0.55 mm). The increase in subsurface hardness primarily results from the pile-up of gliding dislocations under cyclic contact stress, leading to a work hardening effect. It should be noted that the precipitates present in the same region may also play an indirect role in this hardening response. While the direct strengthening contribution from these fine carbides is limited due to their small size and uniform dispersion, they act as obstacles to dislocation motion, thereby promoting dislocation pile-up and entanglement during cyclic deformation. This effect is more pronounced in the 230 °C-tempered specimen, where the greater extent of carbide precipitation leads to more intensive dislocation accumulation and consequently a higher degree of subsurface hardening. This implies that the sample tempered at 230 °C can accommodate a greater number of fatigue-induced dislocations, thereby absorbing and storing more energy from the contact stress.

**Figure 9:**
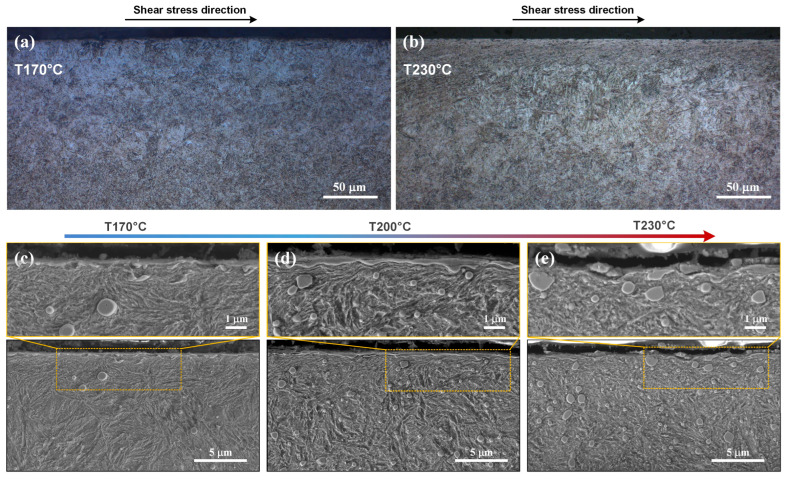
Microstructure in cross profile along normal direction of the fatigue wear tested samples with different tempering at 170 °C (**a**,**c**), 200 °C (**d**), and 230 °C (**b**,**e**).

Figure 9 presents the microstructural characteristics within the near-surface region of the cross-section for samples subjected to 8 million cycles of contact fatigue testing. As shown in Figure 9a,c, the martensitic morphology in the near-surface region of the sample tempered at 170 °C remains largely intact, with no evidence of discernible plastic flow, indicating that significant plastic deformation did not occur prior to the initiation of fatigue spalling. With increasing tempering temperature, as depicted in Figure 9d,e, bands of plastically deformed microstructure gradually appear in the near-surface region of samples tempered at 200 °C and 230 °C. Concurrently, the primary granular carbides exhibit characteristics of both compression and elongation. These observations suggest that under cyclic contact stress, the near-surface martensitic microstructure in these samples undergoes strain hardening first, followed by the initiation and propagation of fatigue cracks, ultimately leading to material removal.

### 3.3. Mechanisms of Strengthening and Wear Resistance

Tempering the high-carbon martensitic microstructure of bearing steel at different temperatures induces several characteristic changes: the decomposition of retained austenite, the precipitation of fine carbides, a reduction in the carbon content of the martensitic matrix, and the stabilization of mobile dislocations. As a soft phase, the decomposition of retained austenite into tempered martensite contributes to an increase in strength. However, due to its limited volume fraction, this contribution to the overall strength enhancement is modest [29]. During tempering, the type of fine carbides precipitated evolves from carbon clusters to transition carbides and finally to stable θ-carbides. Their contribution to strength is primarily through precipitation strengthening via the Orowan dislocation bypassing mechanism [38,39]. The effectiveness of this strengthening diminishes with increasing carbide size; thus, its contribution to the overall strength is also limited. In contrast, the decrease in carbon content within the martensitic matrix drastically reduces its solid-solution strengthening effect [40,41]. It is generally accepted that a reduction of 0.1 wt.% in carbon content decreases the solid-solution strengthening contribution by approximately 300 MPa. Therefore, the weakening of solid-solution strengthening is the primary factor responsible for the overall strength reduction during tempering. Additionally, the low-temperature tempering process facilitates the recovery and stabilization of mobile dislocations retained from the quenching stage, leading to a concurrent reduction in dislocation strengthening [4].

On the other hand, correlating with the changes in compressive mechanical properties, it is reasonable to conclude that after low-temperature tempering, the reduction in solid-solution strengthening and the stabilization of stored dislocations enable more dislocations to undergo slip under compressive stress, with a lower resistance to initiating such movement. This consequently manifests as a lower strain hardening rate. Precisely because of this, strain can accumulate to a larger value under load without undergoing premature fracture caused by stress concentration resulting from dislocation pile-up. Thus, the material exhibits a greater compressive failure strain.

**Figure 10:**
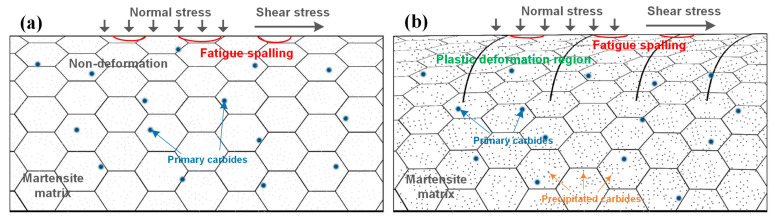
Schematic illustrating the evolution of fatigue-wear damage on the sample cross-section: (**a**) insufficiently tempered; (**b**) adequately tempered at low temperature.

Under cyclic contact stress, the transition from an intact near-surface microstructure to fatigue spalling is, at the micro-scale, fundamentally a process of dislocation slip and progressive pile-up [42,43]. As shown in Figure 10a, for the sample tempered at 170 °C, some retained austenite persists, and the tempering of the high-carbon martensite is incomplete, with no significant carbide precipitation observed. Under cyclic contact stress, the retained austenite in the near-surface region is prone to undergo martensitic transformation. The resulting high-carbon martensite lacks ductility and can become a site for fatigue crack initiation. Furthermore, the insufficiently tempered martensite, due to the pinning effect of solute carbon atoms, cannot accommodate sufficient dislocation slip. This leads to extensive dislocation pile-up over short distances, forming fatigue crack nuclei.

Consequently, the wear cross-section of this sample exhibits only a shallow plastic deformation layer beneath the worn surface, and material removal occurs primarily through fatigue spallation, rather than extensive plastic flow. The limited dislocation slip capability of the insufficiently tempered martensite, caused by the high solid-solution carbon content, restricts the material’s ability to accommodate cyclic strain, thereby promoting earlier fatigue crack initiation and propagation. Under the same stress level, this process occurs extensively, resulting in higher wear mass loss.

However, as the tempering temperature increases to 230 °C, the sample’s microstructure consists predominantly of tempered martensite with evident precipitation of fine carbides, as schematically summarized in Figure 10b based on the experimental observations. Under cyclic contact stress, the well-tempered martensitic matrix can accommodate a greater extent of long-range dislocation slip, exhibiting improved plasticity and toughness. The alternating application of contact stress can be regarded as a process of alternating loading and unloading, where the local stress exceeds the yield strength. Consequently, the entire loading process constitutes a continuous accumulation of slip-induced plastic deformation. Correspondingly, when dislocation slip and pile-up reach a critical limit, a fatigue crack nucleus forms, ultimately leading to the occurrence of fatigue spallation. However, the very process of plastic deformation in the near-surface region indicates an enhanced ability to absorb the strain energy imparted by the contact stress. Therefore, under the same stress level, the occurrence of fatigue spallation is delayed, which manifests as superior resistance to fatigue wear.

## 4. Conclusions

This study investigated the effects of different low-temperature tempering temperatures on the microstructural evolution, compressive mechanical properties, and contact fatigue wear resistance of high-carbon martensitic bearing steel. The corresponding strengthening and wear resistance mechanisms were also discussed. The main conclusions are as follows:

(1) After spheroidizing annealing and quenching, the microstructure of the high-carbon bearing steel consists of high-carbon martensite and retained austenite, containing a high density of dislocations and fine twins. Low-temperature tempering promotes the decomposition of retained austenite into tempered martensite and the precipitation of fine carbides from the martensitic matrix. Higher tempering temperatures lead to more complete decomposition of retained austenite and more pronounced carbide precipitation.
(2) Compared to the as-quenched condition, low-temperature tempering reduces hardness and compressive strength but increases the compressive failure strain, indicating enhanced ductility and toughness. However, with increasing low-temperature tempering temperature, a distinct plastic deformation zone forms in the near-surface region under cyclic contact stress. This is accompanied by a significant reduction in fatigue wear mass loss, demonstrating improved resistance to fatigue wear.
(3) During the low-temperature tempering stage, the decrease in strength is primarily attributed to the reduction in solid-solution carbon content in the martensite matrix, leading to diminished solid-solution and dislocation strengthening. Under cyclic contact stress, the well-tempered martensitic matrix can accommodate more long-range dislocation slip, exhibiting better plasticity and toughness. When dislocation slip and pile-up reach a critical limit, fatigue crack nuclei are considered to form, ultimately resulting in fatigue spallation. The occurrence of plastic deformation in the near-surface region signifies a greater capacity to absorb the strain energy from contact stress. Consequently, under the same stress level, the onset of fatigue spallation is delayed, which corresponds to superior fatigue wear resistance.

## Figures and Tables

**Table 1:** Compressive properties and hardness of tempered samples, including ultimate strength (US), total strain (TS), and product of strength and strain (PSS).

Samples	Processing	US (MPa)	TS (%)	PSS (GPa·%)	Hardness (HV)
Q	Quenching	4250	10.5	35.86	810
T170 °C	Tempering at 170 °C	3566	13.5	41.79	770
T200 °C	Tempering at 200 °C	3480	14.8	45.43	710
T230 °C	Tempering at 230 °C	3168	24.1	68.35	690

## Data Availability

The original contributions presented in the study are included in the article, further inquiries can be directed to the corresponding authors.
